# Antimalarial Efficacy and Antioxidant Activity of *Lophira lanceolata* Stem Bark Ethanol Extract Using *Plasmodium berghei* Induced-Malaria in *Swiss Albino's* Mice

**DOI:** 10.1155/2023/9400650

**Published:** 2023-08-18

**Authors:** Mounvera Abdel Azizi, Noumedem Anangmo Christelle Nadia, Yamssi Cedric, Ngouyamsa Nsapkain Aboubakar Sidiki, Gamago Nkadeu Guy-Armand, Djeussi Doriane Esther, Tientcheu Noutong Jemimah Sandra, Tako Djimefo Alex Kevin, Vincent Khan Payne

**Affiliations:** ^1^Department of Animal Biology, Faculty of Science, University of Dschang, P.O. Box 067, Dschang, Cameroon; ^2^Department of Microbiology, Hematology and Immunology, Faculty of Medicine and Pharmaceutical Sciences, University of Dschang, P.O. Box 96, Dschang, Cameroon; ^3^Department of Biomedical Sciences, Faculty of Health Sciences, University of Bamenda, P.O. Box 39, Bambili, Cameroon; ^4^Department of Animal Organisms, Faculty of Science, University of Douala, P.O. Box 24157, Douala, Cameroon

## Abstract

**Background:**

Malaria remains a major public health problem in the tropical and subtropical regions. This study aimed of investigating the antimalarial and antioxidant activities of ethanol extract of *Lophira lanceolata* stem bark. *Methodology*. The antimalarial activity was determined using the Peter 4-days' suppressive and Rane's curative tests on Swiss albino: these mice were infected with 1 × 10^7^ parasitized red blood cells. The percentage reduction of parasitemia was related to each test, and the liver homogenate was used to assay malondialdehyde, superoxide dismutase, nitrogen monoxide, catalase, and glutathione for the evaluation of oxidative stress. During the curative test, blood was collected for hematological parameters, alanine aminotransferase and aspartate aminotransferase to evaluate liver function.

**Result:**

The ethanol extract of *L. lanceolata* showed a dose-dependent suppressive activity with the highest suppression of 88.22% at 500 mg/kg. Suppression produced by the extract was not significantly higher than that of the reference drug with 96.1%. Similarly, the extract at doses 125, 250, and 500 mg/kg showed significant decreases (*P* < 0.05) in a dose-dependent manner during the curative test. The ethanol extract *of L. lanceolata* caused a reduction of tissue markers, such as hepatic oxidative stress, as it increased the enzymatic activity of antioxidant enzymes.

**Conclusion:**

The ethanol extract of *L. lanceolata* possesses both antimalarial and antioxidant activities. However, further *in vivo* toxicity tests are required to guarantee their safety.

## 1. Background

Malaria is an infectious disease caused by parasites of the genus *Plasmodium* and transmitted by female *Anopheles* mosquitoes during blood meals [1]. It is the most common parasitic disease in the world, especially in tropical and subtropical regions. This disease particularly affects children less than five years old and pregnant women. The number of malaria cases was estimated at 229 million with 409,000 deaths, 94% in the African region [2]. In Cameroon, this disease is the main cause of consultation and the leading cause of morbidity and mortality [3]. Previous *in vitro* research work conducted by Abdel Azizi et al. [4] and Guy-Armand et al. [5] demonstrated that there is a close relationship between malaria and oxidative stress. According to these authors, during malaria infection, there is an overproduction of free radicals, which are toxic to the host organism. Hence, there is a pressing need to search for alternative drugs having both antimalarial and antioxidant activities. From year to year, the morbidity and mortality of this disease continues to increase due to the resistance of the parasite to the available antimalarial drugs, none availability of the antiplasmodial drugs to the local population, and resistance of the female *Anopheles* mosquitoes to available insecticides [6]. Furthermore, malaria is accompanied by inflammatory and oxidative processes induced by the parasite, and the febrile state is the first clinical sign following successive bursting of parasitized erythrocytes [7].

Since ancient times, medicinal plants have been a source of remedy for human health. Today, herbal medicine remains the cheapest and most accessible form of remedying a vast majority of the population [8]. In this respect, *L. lanceolata* belonging to the Ochnaceae family could be an interesting plant. *Lophira lanceolata* is a medicinal plant used by the local population in Cameroon for the treatment of fever, bacterial infection, and digestive issues. Fermented palm wine (ethanol) or infusion is used by traditional practitioners for the preparation of medicinal remedies in Cameroon. Abdel Azizi et al. [4] reported the *in vitro* antiplasmodial, cytotoxicity, and antioxidant activities of *L. lanceolata.* The study of Abdel Azizi et al. [4] reported a moderate *in vitro* antiplasmodial activity of the aqueous extract according to the classification of Kumari et al. [9] with IC_50_ = 56.365 and 51.36 *μ*g/ml for sensitive and resistant strain, respectively, and resistance index of 0.09 (IR = 0.09). The ethanol extract showed interesting plasmodial activity, revealing an IC_50_ of 31.865 and 24.515 *μ*g/ml for the sensitive and resistant strains, respectively, and a resistance index of 0.01 (IR = 0.01). Cédric et al. [10] recently demonstrated the antihelminthic activity of *L. lanceolata*. Similarly, according to Sinan et al. [11], the chemical profiling of *L. lanceolata* confirmed the presence of lanceolatins and lophirones. Higher levels of total phenolics (156.42 mg gallic acid equivalent/g for maceration-water), phenolic acids (165.84 mg caffeic acid equivalent/g for maceration-water), and flavonols (101.51 mg catechin equivalent/g for soxhlet-methanol) were observed in the stem bark extracts. Nadia et al. [12] demonstrated that an extract can be active *in vitro* but inactive *in vivo* due to certain biochemical processes, such as biotransformation and bioavailability, of the extract in the host organism. It is, therefore, of paramount importance to assess *in vivo* antimalarial and antioxidant activities of *L. lanceolata* to confirm their activities. The overall aim of this research was to investigate the antimalarial and antioxidant activities of the ethanol extract of *L. lanceolata* stem bark to justify its usage by traditional healers in Cameroon and ascertain its potential as an antimalarial drug.

## 2. Methodology

### 2.1. Plant Collection and Identification

The stem bark of *L. lanceolata* was harvested in the Foumbot subdivision, Noun Division, West Region of Cameroon. It was further identified at the National Herbarium with voucher number 4002B.

### 2.2. Animal Organism

Swiss albino mice aged 8 weeks maximum and weighing 20–25 g were obtained from the Animal Facility of the Faculty of Agronomy and Agricultural Sciences at the University of Dschang. Mice were maintained under standard and constant laboratory conditions with free access to food and water. Animal welfare requirements were rigorously taken into account during the experiments according to the recommendations of the Ethics Committee.

### 2.3. Preparation of the Ethanol Extract of *L. lanceolata* Stem Barks

The ethanol extract was obtained using the method described by Cedric et al. [10]. Briefly, 100 g of *L. lanceolata* powder was introduced into 1 l of 95% ethanol and remained in the solvent for 72 hours with occasional stirring. The mixture was filtered using Whatman number 1 filter paper. The extract was obtained from the evaporation of the solvent in an oven at 40°C. The ethanol extract was stored in a refrigerator at 4°C for further usage.

#### 2.3.1. Extraction Yield

The percentage yield of *L. lanceolata* was calculated using the following formula:

(1) $$\begin{array}{c} \text{Percentage yield}=\frac{\text{Weight of dried extract}}{\text{Weight of dried plant sample}}\times 100. \end{array}$$

### 2.4. Malaria Parasite

The NK65 strain of *Plasmodium berghei* was obtained from BEI-Resources (Manassas, VA USA) and maintained by sub-passage in laboratory mice.

### 2.5. Preparation of the Inoculum

The method described by Nadia et al. [13] was used for the preparation of the inoculum. Briefly, the contents of the vial were inoculated intraperitoneally into two female Swiss mice, weighing approximately 25 ± 3 g. Blood was analyzed every two days to assess the parasitemia. For this, a drop of blood was collected from the mouse tail to prepare a blood smear. Once one of the two mice had parasitemia ≥30%, the mouse was sacrificed, and the blood was collected and introduced into an Eppendorf tube containing heparin. This blood was diluted with Phosphate-buffered saline. Then, each experimental mouse received intraperitoneally 200 *μ*L containing 1 × 10^7^ parasitized red blood cells (RBCs).

### 2.6. Preparation of Gavage Solution

The method described by Sidiki et al. [14] was used for the preparation of the gavage solution. Three hundred milligrams of the ethanol extracts were mixed with dimethylsulfoxide (DMSO) to facilitate dissolution. This mixture was dissolved in 20 mL of distilled water to be administered to experimental animals orally, at doses 125, 250, and 500 mg/kg The volume of each of these substances was administered to each animal and calculated according to the formula:

(2) $$\begin{array}{c} \text{Volume administered }\left(\text{ml}\right)=\frac{\text{Animal body weight }\left(\text{kg}\right)\times \text{dose of extract}}{\text{Concentration of extract solution }\left(\text{mg}/\text{ml}\right)}. \end{array}$$

### 2.7. Antimalarial Activity

#### 2.7.1. Peter Four-Day's Suppressive Test

**Figure 1:**
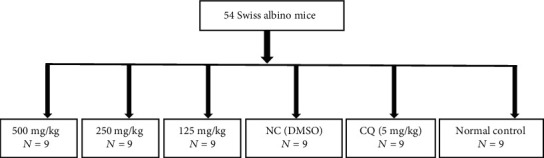
Peter 4 days' suppressive test experimental design.

The method described by Knight and Peters [15] was adopted with slight modifications. Briefly, 45 Swiss albino mice out of 54 were infected intraperitoneally with 1 × 10^7^ parasitized RBCs and were divided into 6 groups, of 9 mice each (Figure 1). Three hours after infection, the extracts and chloroquine (CQ) were administered orally to the animals. These mice were treated at 24-hour intervals for 4 days (D0, D1, D2, and D3). From the 5th to the 9th day (D4–D8), the parasitemia of each mouse was evaluated. From the 9th to the 30th day, the mice were observed and the average survival rate was calculated and recorded according to the following formula [16].

(3) $$\begin{array}{c} \text{Mean parasitemia}=\frac{\text{Number of parasitized RBCs}}{\text{Total number of counted RBCs}}\times 100. \end{array}$$

The percentage of suppression was calculated according to the following formula [14]:

(4) $$\begin{array}{c} \%\text{Suppression}=1-\frac{\text{Parasitemia in test group}}{\text{Parasitemia of the negative control}}\times 100. \end{array}$$

#### 2.7.2. Curative Test

The method described by Nadia et al. [12] was used. Briefly, 45 Swiss albino mice out of 54 were infected intraperitoneally with 1 × 10^7^ parasitized RBCs and were divided into 6 groups, of 9 mice each. The animals were given the extract: 1% DMSO, reference drug, and distilled water as previously indicated above. They were treated on D4, D5, D6, and D7. The parasitemia of all experimental groups was evaluated on day what except for the normal control group [17]. The parasitemia reduction rate was calculated as follows [14].

(5) $$\begin{array}{c} \text{Parasitemia reduction rate}=\frac{\text{Parasitemia of control}-\text{parasitemia of the test group}}{\text{Parasitemia of control}}\times 100. \end{array}$$

The mean survival rate (MSR) of each group was determined over a period of 30 days according to the following formula [14]:

(6) $$\begin{array}{c} \text{Mean survival rate}=\frac{\text{Number of days survived }}{\text{Total of days }\left(30\right)}\times 100. \end{array}$$

### 2.8. Antioxidant Activity and Biochemical Parameters

Mice were sacrificed on the 10th-day, and blood and liver samples were collected. Blood was collected for evaluation of biochemical parameters, such as alanine aminotransferase (ALAT) and aspartate aminotransferase (ASAT) using the Dutch Diagnostic Kit. The liver was collected for evaluation of parameters, such as malondialdehyde (MDA), glutathione (GSH), nitrogen monoxide (NO), protein, superoxide dismutase (SOD), and catalase (CAT), using a spectrometer (BIOSE BK-D560). The hematological parameters were equally evaluated using a hematological analyzer.

### 2.9. Ethical Approval

All Authors hereby declare that “Principles for the Care of Laboratory Animals” (NIH publication N0 85-23, revised 1985) have been followed as well as specific National Laws, where applicable [18]. All experiments were reviewed and approved by the Department of Animal Biology, Faculty of Sciences, University of Dschang.

### 2.10. Study Quality Control Measures

The study of Onyeto et al. [19] on the sub-acute toxicity profile of methanol leaf extract of *L. lanceolata* (Ochnaceae) in rats suggests that the leaf extract of *L. lanceolata* is safe and well-tolerated and devoid of deleterious effects on the vital organs.

### 2.11. Statistical Analysis

Data were recorded in Excel and then transferred to the Graph Pad Prism software version 8.4.2 for analysis, and the results were presented as the mean ± SD using Analysis of variance (ANOVA) one way and multiple comparison tests, values were considered significant at *p* < 0.05.

## 3. Results

The yield obtained after extraction with ethanol solvents from 100 g of *L. lanceolata* stem bark powder was 8.15%.

### 3.1. Antimalarial Activity

#### 3.1.1. Parasitemia and Suppression Rate

Table 1 shows the suppressive effect, level of parasitemia, and mean survival rate. Observations from Table 1 indicates that parasitemia and suppression rate, respectively, are dose-dependent and had a negative influence on the development of *P. berghei* by reducing its parasitemia.

**Figure 2:**
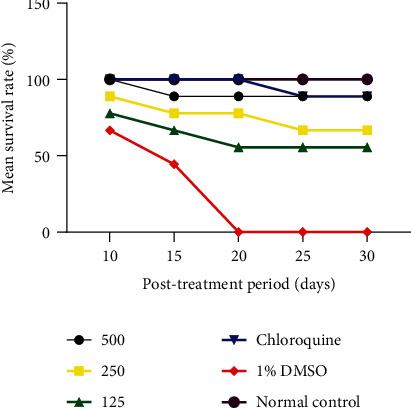
Mean survival rate of experimental animals during the post-treatment period of the Peter 4 Days Suppressive.

*(1) Mean Survival Rate of Animals during the Post-Treatment Period of Peter 4 Days Suppressive*.  Figure 2 shows the cumulative number and mean survival rate (%) of the experimental animals after stopping treatment at different doses. It appears from Figure 2 that the animals, which received CQ 5 mg/kg, and the extract at a dose of 500, 250, and 125 mg/kg survived for 30 days post-treatment, unlike the animals in the negative control group (DMSO), which shows a survival rate of 0% from day 20. The survival rate is proportional to an increase in dose, the higher the dose the higher the survival rate. A significant difference is observed between the different doses and the DMSO-treated group from day 20 (*P* < 0.05).

#### 3.1.2. Curative Activity of the Extract on the Development of *Plasmodium berghei*

**Figure 3:**
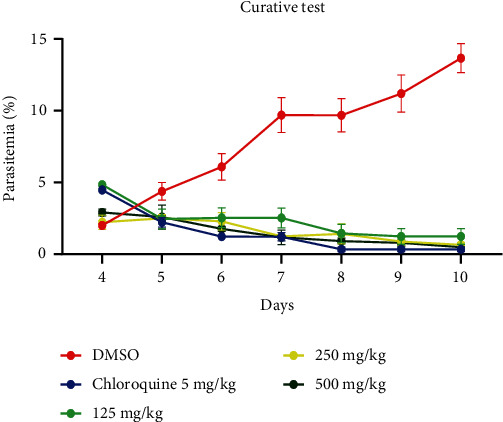
Curative effects of different doses of ethanol extract of *L. lanceolata* stem bark in curative test.

Figure 3 shows the curative effects of different doses of the extract and it follows from the analysis that the doses 125, 250, and 500 mg/kg had a negative influence on the development of *P. berghei* by reducing its parasitemia and were statistically different (*P* < 0.05) as compared with the 1% DMSO group. However, no significant difference (*P* > 0.005) was observed among the tested groups and between the tested groups and CQ 5 mg/kg.

**Figure 4:**
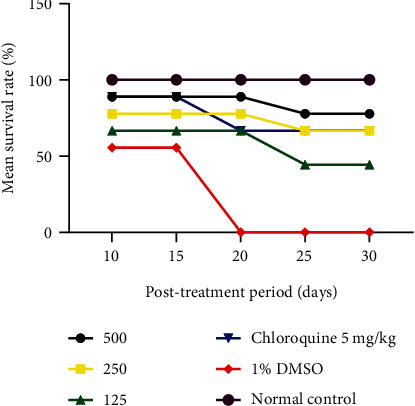
Mean survival rate of experimental animals during the post-treatment period of the curative test.

*(1) Mean Survival Rate of Animals during the Post-Treatment Period*.  Figure 4 shows the MSR (%) of the animal during the post-treatment period of the curative test. Table 1 shows that animals, which received CQ 5 mg/kg, and the extract at a dose of 500, 250, and 125 mg/kg survived for 30 days post-treatment, unlike the animals of the DMSO-treated group, which showed a 0% survival on day 20. From the analysis of this Table 1, it is noted that the higher the dose, the higher the survival rate. A significant difference was observed between the different doses and 1% DMSO from the 2 0th day.

*(2) Effect of Treatment or Haematological Parameters*.  Table 2 shows the effect of *L. lanceolata* extract on hematology parameters. From this Table 2, it is observed that white blood cells, hemoglobin level, and hematocrit level did not undergo any fluctuation in all doses (*P* > 0.05), unlike elements, such as lymphocytes, monocytes, and RBCs, which varied significantly. Extract of doses 500 and 250 mg/kg compared with 1% DMSO revealed a significant difference (*P* < 0.05) for lymphocytes. However, for monocytes, the results were statistically different between the different doses and the negative control, contrary to RBCs, in which a significant difference was observed in the CQ-group and the dose 500 mg/kg compared with the negative control (*P* < 0.05).

### 3.2. *In Vivo* Antioxidant Activity of *L. lanceolata* Extract

#### 3.2.1. Effect of *L. lanceolata* Stem Bark Ethanol Extract on Oxidative Stress Parameters

Table 3 shows the effect of *L. lanceolata* on oxidative stress. From the analysis of this Table 3, it is noted that the level of catalase in the DMSO-treated group was low (2.25) compared with the other treated groups. The group to which extract of 500 and 250 mg/kg was given showed an increase in catalase levels of 0.3505 and 0.3062, respectively. The same observation was noticed for SOD where the highest level was obtained for the group treated groups with extract compared with the negative control. The extract-treated dose of 125 mg/kg presented the highest level of SOD (6.910). The ethanol extract administered showed a decrease in hepatic NO at 500 and 250 mg/kg compared with the DMSO-treated group. The groups 125 mg/kg, CQ, and DMSO showed an increase in hepatic NO with no significant difference observed (*P* > 0.05).

MDA revealed no significant difference (*P* > 0.05) between 500 mg/kg, 250 mg/kg, and 1% DMSO groups. However, at the dose of 125 and 5 mg/kg of CQ a statistically significant difference was observed (*P* < 0.05).

It follows from the analysis of this Table 3 that infection resulted in an increase in GSH compared with the normal control. The administration of extracts at different doses resulted in a decrease in the rate of GSH compared with the negative control.

### 3.3. Biochemical Parameters

Table 4 shows the effect of *L. lanceolata* on biochemical parameters. We noticed from this Table 4 that ASAT varies concerning dose. Between 500 mg/kg, 250 mg/kg, and CQ, there was no significant difference with 1% DMSO, whereas a statistically significant difference was observed between the treated group 125 mg/kg and 1% DMSO (*P* < 0.05). ALAT varies according to dose. With extract 500 and 250 mg/kg groups had no significant difference concerning DMSO, whereas a statistically significant difference was observed between the neutral control, 125 mg/kg, and CQ group (*P* < 0.05).

## 4. Discussion

In the western region of Cameroon, the traditional healers used the stembark of *L. lanceolata* to treat many diseases like fever, helminthiasis, bacteria, and malaria. The ethanol extract showed a suppressive activity on the development of *P. berghei in vivo*, which was previously tested *in vitro* by Abdel Azizi et al. [4] and demonstrated promising antiplasmodial activity according to different concentrations. This result corroborates those obtained by De Carvalho and Jacobs [20] who showed that the leaves of *L. lanceolata* had a suppressive percentage of 88% compared with the reference drug (CQ). These results are in contradiction with that of Onyeto et al. [21] who obtained a suppressive activity of 76% with the leaves of *L. lanceolata*, and treatment was not concentration dependent. This difference could be due to the phytochemical constituents of the plant, climatic factors, the part of the plant used, and even the geographical location. After the infection of the mice with *P berghei*, we found a slight and rapid multiplication. According to Nadia et al. [12], an increase in the number of parasites in the blood of mice 4 days after infection testifies to the establishment of infection. The reference drug used in this work exerts a suppression of 96.10% at a dose of 5 mg/kg. Kamei et al. [22] showed that when a standard antiplasmodial drug is used on a mouse infected with *P. berghei*, it suppresses the parasite to an undetectable level, which is in line with our study. These percentages of suppression show that the secondary metabolites in the extract may have acted by preventing the diffusion and migration of the parasites (merozoites) into the bloodstream [23]. The suppressive test showed that parasite eliminations were significantly proced on the day 3. This result is similar to that found by Onyeto et al. [21], who worked on the leaves of *L. lanceolata.* According to Abdel Azizi et al. [4] this plant contains secondary metabolites, such as flavonoids, polyphenols, alkaloids, sterols, triterpenoids, anthocyanins, and many other constituents, that may have acted by preventing the installation of the parasite and clinical symptoms, suggesting the possibility of inducing healing, and this action was observed with all doses used in this work.

The ethanolic extract of *L. lanceolata* showed the ability to inhibit parasite growth after its installation at different doses. Depending on the doses, a considerable decrease in parasitemia was observed with increasing doses in mice. These results are similar to those obtained by Etkin [24], who showed that the reduction in parasitemia is dose-dependent. These results also corroborate those obtained by Falade et al. [25] in Nigeria showed *in vitro* and *in vivo* antiplasmodial activity of the *Lophira alata* plant from the same family as *L. lanceolata*. A dose-dependent chemosuppression of parasitemia and a prolongation of the survival of infected animals were observed in the treated groups. This prophylactic and curative activity may be attributed to secondary metabolite in the ethanol extract of *L. lanceolata,* which was found by Abdel Azizi et al. [4] who worked on *in vitro* antiplasmodial, cytotoxicity, and antioxidant activities of *L. lanceolata* (Ochnaceae) used in Cameroon to treat malaria. The antimalarial activity of *L. lanceolata* could be due to the presence of a secondary metabolite that may have acted by preventing DNA and RNA synthesis by blocking the dihydrofolate reductase and dihydropteroate in the cytosol, by targeting the cytochrome bc1 complex, inhibits the parasitic electron transport chain and, respectively, the dihydroorotate dehydrogenase linked to the respiratory chain and implicated in the pyrimidine nucleotides biosynthesis [21].

A significant survival rate of mice after treatment was observed. These results could be due to the effectiveness of the phytochemicals that *L. lanceolata* possesses. The highest dose used (500 mg/kg) reveals almost the same ability to inhibit the parasite compared with the reference drug. These results differ from those obtained by Onyeto et al. [21] who worked on the leaves of *L. lanceolata* plants in Nigeria. These differences in results could be explained by several parameters, among others, the disparity of the parasitic strains tested, the methods of extraction, the harvesting period, and even the edaphic properties, which are parameters, which may influence the chemical constituents of plants. Kabata-Pendias [26] has shown that the phytochemical constituents of plants differ according to soil and climate, but that these constituents have an important role to play through the various pharmaceutical properties they contain.

Regarding the biochemical parameters in this study, ASAT and ALAT vary concerning dose. The higher level of ASAT and ALAT in the negative control compared with the treated groups suggest the hepatoprotective effects of the plants. This activity could be attributed to their content of bioactive phenolic compounds of medium polarity and apolar compounds, such as glycolysed flavonoids and aglycones, which are extracted by alcohols more than by water, which explains the greater antioxidant power and hepatoprotective effect of ethanol extracts than of aqueous extracts. These results are similar to those obtained by Zerargui [27] who worked on antioxidant activity of *Tamus communis* L. root extracts and the characterisation of bioactive substances.

White blood cells play an important role in the immune defense against foreign bodies, generally through leukocytosis and the production of antibodies. When leukocytes increase in the body, they signal the presence of a foreign body (parasite). In our study, white blood cell, hemoglobin level, and hematocrit level did not undergo any significant increase at all doses (*P* > 0.05). These results are similar to those obtained by Nadia et al. [12] who worked on *in vitro* antiplasmodial activity and cytotoxicity of extracts and fractions of *Bidens pilosa* in Cameroon. These results could be due to the chemical constituents of the plant, which can inhibit the development of parasites and in turn prevent the destruction of white blood cells, hemoglobin levels, and hematocrit levels. Certain properties, such as the flavonoids, found in the plant, have anti-inflammatory properties and are capable of modulating the functioning of the immune system by inhibiting the activity of enzymes that may be responsible for inflammation. They can also modulate the adhesion of monocytes during inflammation by inhibiting the expression of inflammatory mediators.

Endogenous enzymatic antioxidants, such as SOD, CAT, and non-enzymatic, such as reduced GSH responsible for detoxifying the body of harmful free radicals in the liver and kidneys [28] .*L. lanceolata* exhibits potent antioxidant power against the oxidative hepatic cell, where it significantly decreased the elevated hepatic levels of MDA and consequently increased the level of GSH and the enzymatic activities of the antioxidant defense system, such as the activity of CAT and SOD. These results are similar to those observed by Bona et al. [28] who worked on effect of antioxidant treatment on fibrogenesis in rats with carbon tetrachloride-induced cirrhosis. These results could be due to the antioxidant effect of *L. lanceolata* and its ability to improve oxidative damage to the liver. Moreover, the maintenance of these markers almost at normal levels, in comparison with the control group, reveals that the species of the genus *Lophira* have an antioxidant capacity *in vivo*, in a dose-dependent manner, thus increasing liver function, which can be attributed to its bioactive secondary metabolites, such as phenolic compounds. Furthermore, the antioxidant activity of the compounds has been attributed to various mechanisms, including the prevention of chain initiation and the binding of catalysts to transition metal ions, which are elements that are harmful to the hepatocyte cell.

## 5. Conclusion

In this study, which aimed to demonstrate the activity of *L. lanceolata* on the development of *Plasmodium berghei*, it emerged that the ethanol extract has a promising effect on the development of the asexual phase of the parasite and has the potential to reduce oxidative stress *in vivo.* However further *in vivo* toxicity tests are necessary to confirm their safetyness.

## Figures and Tables

**Table 1:** Suppressive effect and level of parasitemia produced by ethanol extract of *L. lanceolata* stem bark in the 4-day suppressive test.

Treatment	Dose (mg/kg)	Parasitemia level	% Suppression
1% DMSO	0	67.20 ± 9.213^b^	0.00
Chloroquine	5	2.620 ± 1.365^a^	96.10
	125	9.608 ± 3.922^a^	85.71
Extract	250	9.110 ± 6.102^a^	86.44
	500	7.910 ± 4.585^a^	88.22

Values are presented as mean ± SD. ^a,b^Values carrying the same superscript letter are not significantly different at *P* < 0.05.

**Table 2:** Effect of *L. lanceolata* stem bark extract on hematology parameters of *P. berghei*-infected mice.

Treatment	Doses (mg/kg)	WBC (*μ*l)	HGB (g/dl)	HCT (%)	LYM	MON	RBC (×10^12^/l)
Extract	500	9.361 ± 0.3672^b^	13.10 ± 0.9644^b^	41.13 ± 2.743^b^	31.63 ± 3.450^a^	8.378 ± 1.323^b^	61.36 ± 3.726^a^
	250	8.544 ± 1.335^b^	13.43 ± 2.761^b^	41.53 ± 8.673^b^	59.30 ± 2.452^a^	14.02 ± 2.583^b^	30.81 ± 7.825^b^
	125	9.877 ± 1.365^b^	13.63 ± 1.270^b^	42.80 ± 3.604^b^	52.59 ± 2.090^b^	10.86 ± 1.564^b^	35.97 ± 7.794^b^
Chloroquine	5	7.155 ± 1.013^b^	11.04 ± 1.500^b^	32.22 ± 3.950^b^	73.34 ± 2.682^a^	14.28 ± 1.185^b^	14.73 ± 2.916^a^
1% DMSO	0	6.274 ± 1.197^b^	9.700 ± 1.997^b^	30.81 ± 6.848^b^	44.64 ± 6.950^b^	19.30 ± 3.407^a^	29.42 ± 5.295^b^
NC	0	7.610 ± 0.7000^b^	12.99 ± 1.483^b^	37.77 ± 9.103^b^	54.33 ± 4.153^b^	10.66 ± 2.786^b^	35.01 ± 3.693^b^

Values are presented as mean ± SD. WBC: white blood cell; HGB: hemoglobin; HCT: hematocrit; LYM: lymphocyte; MON: monocyte; RBC: red blood cell; DMSO: dimethyl sulfoxide; NC: normal control. ^a,b^Values carrying the same superscript letter are not significantly different at *P* < 0.05.

**Table 3:** Effect of *L. lanceolata* stem bark extract on oxidative stress parameters of *P. bergei*-infected mice.

Treatment	Doses (mg/kg)	Catalase	Superoxide dismutase	Nitrogen monoxide	Malondialdehyde	Glutathione
Extract	500	0.3505 ± 0.1123^a^	2.498 ± 0.6573^b^	11.40 ± 0.9524^a^	1.193 ± 0.5668^b^	28.66 ± 1.588^b^
	250	0.2617 ± 0.1027^a^	2.321 ± 0.6378^b^	10.63 ± 3.339^a^	1.512 ± 0.4271^b^	18.34 ± 0.8993^a^
	125	0.3062 ± 0.02117^a^	6.910 ± 1.118^a^	18.52 ± 7.827^b^	1.169 ± 0.2337^b^	18.74 ± 2.033^a^
Chloroquine	5	0.2608 ± 0.1184^a^	5.717 ± 0.7881^b^	19.47 ± 7.460^b^	0.1667 ± 0.4174^a^	11.05 ± 2.327^a^
1% DMSO	0	0.2599 ± 0.09098^a^	1.295 ± 0.7436^b^	8.954 ± 1.810^a^	0.4758 ± 0.2023^a^	29.92 ± 2.129^b^
NC	0	0.2393 ± 0.07530^a^	6.654 ± 1.425^a^	26.26 ± 5.649^b^	1.572 ± 0.4401^b^	13.86 ± 1.728^a^

Values are presented as mean ± SD. DMSO: dimethyl sulfoxide; NC: normal control. ^a,b^Values carrying the same superscript letter are not significantly different at *P* < 0.05.

**Table 4:** Effect of *L. lanceolata* stem bark ethanol extract on the biochemical parameters.

Treatment	Doses (mg/kg)	ASAT	ALAT	Protein
Extract	500	62.66 ± 10.66^b^	56.89 ± 5.093^b^	62.17 ± 5.585^a^
	250	58.56 ± 9.223^b^	42.49 ± 3.201^b^	67.07 ± 10.85^a^
	125	6.839 ± 0.7275^a^	16.73 ± 1.892^a^	60.94 ± 4.931^a^
Chloroquine	5	70.03 ± 12.80^b^	21.15 ± 1.552^a^	65.88 ± 10.31^a^
1% DMSO		75.63 ± 21.48^b^	52.04 ± 2.280^b^	69.46 ± 8.137^a^
NC		59.56 ± 8.253^b^	22.70 ± 0.5820^a^	66.16 ± 0.8635^a^

Values are presented as mean ± SD. DMSO: dimethyl sulfoxide; NC: normal control. ^a,b^Values carrying the same superscript letter are not significantly different at *P* < 0.05.

## Data Availability

All data generated and analysed are included in this research article.

## Additional Points

*Limitations of the study*. The limitations of the study include the lack of toxicity tests, phytochemical screening, and the isolation of the active compounds responsible for the antimalarial activity.
